# Increased frequency of CCR4+ and CCR6+ memory T-cells including CCR7+CD45RA^med ^very early memory cells in granulomatosis with polyangiitis (Wegener's)

**DOI:** 10.1186/ar3794

**Published:** 2012-04-10

**Authors:** Ursula Fagin, Silke Pitann, Wolfgang L Gross, Peter Lamprecht

**Affiliations:** 1 Department of Rheumatology, Vasculitis Center UKSH and Clinical Center Bad Bramstedt, University of Lübeck, Ratzeburger Allee 160, 23538 Lübeck, Germany

## Abstract

**Introduction:**

Chemokine receptors play an important role in mediating the recruitment of T cells to inflammatory sites. Previously, small proportions of circulating Th1-type CCR5+ and Th2-type CCR3+ cells have been shown in granulomatosis with polyangiitis (GPA). Wondering to what extent CCR4 and CCR6 expression could also be implicated in T cell recruitment to inflamed sites in GPA, we investigated the expression of CCR4 and CCR6 on T cells and its association with T cell diversity and polarization.

**Methods:**

Multicolor flow cytometry was used to analyze CCR4, CCR6, and intracellular cytokine expression of T cells from whole blood of GPA-patients (*n *= 26) and healthy controls (*n *= 20). CCR7 and CD45RA were included for phenotypic characterization.

**Results:**

We found a significant increase in the percentages of circulating CCR4+ and CCR6+ cells within the total CD4+ T cell population in GPA. In contrast, there was no difference in the percentages of CD8+CCR4+ and CD8+CCR6+ T cells between GPA and healthy controls. CCR4 and CCR6 expression was largely confined to central (T_CM_) and effector memory T cells (T_EM_, T_EMRA_). A significant increase in the frequency of CCR4+ and CCR6+ T_EMRA _and CCR6+ T_CM _was shown in GPA. Of note, we could dissect CCR4 and CCR6 expressing CCR7+CD45RA^med ^very early memory T cells (T_VEM_) from genuine CCR7+CD45RA^high ^naïve T cells lacking CCR4 and CCR6 expression for peripheral tissue-migration within the CCR7+CD45RA+ compartment. The frequencies of CCR4+ and CCR6+ T_VEM _were also significantly increased in GPA. An increased percentage of IL-17+ and IL-22+ cells was detected in the CCR6+ cell subsets and IL-4+ cells in the CRR4+ cell subset when compared with CD4+ cells lacking CCR4 and CCR6 expression.

**Conclusions:**

Increased frequencies of circulating CCR4+ and CCR6+ memory T cell subsets including hitherto unreported T_VEM _suggest persistent T cell activation with the accumulation of CCR4+ and CCR6+ cells in GPA. CCR4 and CCR6 could be involved in the recruitment of T cells including cytokine-producing subsets to inflamed sites in GPA.

## Introduction

T cells display considerable heterogeneity in terms of phenotype, function, and anatomical distribution. Whereas naïve T cells represent a relatively homogenous population, primed T cells acquire effector functions and differentiate into distinct effector and memory subsets. Whereas naïve and central memory T cells home to secondary lymphoid organs to mount antigen-driven proliferative responses, effector memory T cells migrate into peripheral tissues to display immediate effector functions such as cytokine production or cytotoxicity or both [1,2]. The process of T cell recruitment from blood into tissue is controlled by adhesion molecules, in which chemokine receptors have an important role [2].

Previously, we showed that a small proportion of circulating memory T cells displays T-helper cell 1 (Th1)-type CC chemokine receptor (CCR) 5 and Th2-type CCR3 expression in granulomatosis with polyangiitis (GPA) [3]. GPA is a rare chronic inflammatory disorder of unknown etiology and is characterized by necrotizing granulomatosis of the upper or lower respiratory tract or both and a systemic autoimmune vasculitis preferentially affecting pulmonary and renal small vessels. The vasculitis is associated with highly specific anti-neutrophil cytoplasmic autoantibodies to proteinase 3 (PR3-ANCA) [4]. T cells are abundant in inflammatory lesions in GPA. CCR5, CCR3, and their chemokine ligand CCL5 (regulated upon activation in normal T cells, expressed and secreted, or RANTES) are expressed in granulomatous lesions of the respiratory tract. These studies suggested that CCR5 and CCR3 could be involved in the recruitment of interferon-gamma (IFNγ)-producing and tumor necrosis factor-alpha-producing Th1- and interleukin (IL)-4-producing Th2-type cells to inflammatory sites in GPA [5-7]. More recently, IL-17-producing PR3-specific Th17 cells have been implicated in the maintenance of chronic inflammation and autoimmunity in GPA [8-10]. CCR4^+ ^T cells have been reported to secrete IL-4, whereas CCR6^+ ^cells produce IL-17 [11,12]. To investigate the extent to which the chemokine receptors CCR4 and CCR6 could be implicated in T-cell recruitment in GPA, we analyzed CCR4 and CCR6 expression on circulating T cells, assigned CCR4- and CCR6-expressing cells to the respective memory cell subsets, and determined the cytokine production of CCR4^+ ^and CCR6^+ ^T cells.

## Materials and methods

### Study population

Patients fulfilled the American College of Rheumatology criteria and the Chapel Hill Consensus Conference definition for GPA, respectively [13,14]. Disease activity was recorded in accordance with European League Against Rheumatism recommendations (Table 1) [15]. All patients and controls provided informed consent. The study was approved by the local ethics committee (#07-059).

**Table 1:** Clinical and laboratory characteristics of patients with GPA and healthy controls

	Patients with GPA in remission	Patients with active GPA	Healthy controls
Number	12	14	20
Age in years, mean (range)	58 (38-83)	55 (27-77)	51 (21-78)
Sex, male/female	10/2	10/4	11/9
BVAS V3.0, mean (range)	0	10 (4-21)	
CRP in mg/L, mean (range)	8 (1-17)	33 (1-86)	
PR3-ANCA, positive/negative	9/3	14/0	
Methotrexate, number	9	5	
Azathioprine, number	1	0	
Leflunomide, number	2	0	
Cyclophosphamide, number	0	5	
Prednisolone, number	12	10	
Prednisolone dosage in mg	6.1 ± 0.8	11.9 ± 1.9^a^	
No treatment	0	4	

Remission was defined as BVAS V3.0 (Birmingham Vasculitis Activity Index version 3.0) of 0. Disease activity in patients either with first manifestation or relapsing was defined as BVAS V3.0 of at least 1 (15). Prednisolone dosage is expressed as mean ± standard error of the mean. ^a^*P *< 0.05 by Mann-Whitney *U *test. CRP, C-reactive protein; GPA, granulomatosis with polyangiitis; PR3-ANCA, anti-neutrophil cytoplasmic autoantibodies targeting proteinase 3.

### Antibodies used for flow cytometry

The following antibodies were used in different combinations: Pacific blue (PB)-conjugated anti-CD3, PB- or phycoerythrin (PE)-conjugated anti-CD4, peridinin chlorophyll protein (PerCP)- or allophycocyanine-cyanine dye 7 (APC-Cy7)-conjugated anti-CD8, fluorescein isothiocyanate (FITC)-conjugated anti-CD45RA, Alexa Fluor 647-conjugated anti-CCR7, PE-Cy7- and PE-conjugated anti-CCR4, PE-conjugated anti-CCR6, APC-Cy7-conjugated anti-IFNγ, PE-Cy7-conjugated anti-IL-4, and Alexa Fluor 488-conjugated anti-IL-17a from eBioscience (Frankfurt, Germany) and APC-conjugated anti-IL-22 from R&D Systems (Wiesbaden, Germany). Appropriate isotype controls were included in the experimental setup. All antibodies (unless indicated otherwise) were purchased from BD Biosciences (Heidelberg, Germany).

### Surface marker and intracellular cytokine staining

Flow cytometry was performed to characterize T cell populations at the single-cell level. Staining of cellular surface markers was performed by using freshly collected whole blood (Li-heparin) as described earlier [3]. Briefly, previously determined optimal concentrations of fluorochrome-conjugated monoclonal antibodies for cell surface antigens were added to 100 μL of whole blood and incubated 45 minutes in the dark at 4°C. Subsequently, erythrocytes were lysed by the addition of FACS (fluorescence-activated cell sorting) Lysing Solution (BD Pharmingen, Heidelberg, Germany). After incubation for another 10 minutes in the dark at room temperature, cells were washed twice with phosphate-buffered saline/0.01% bovine serum albumin and immediately analyzed by FACS.

For intracellular cytokine staining, freshly collected whole blood was stimulated with phorbol myristate acetate (Sigma, Munich, Germany) (10 ng/mL) and ionomycin (Sigma) (1 μg/mL) for 4 hours at 37°C in a humidified atmosphere with 5% CO_2_. Brefeldin (Sigma) (10 μg/mL) was added at the beginning of the stimulation to inhibit cytokine secretion. After staining for surface antigens and lysing of erythrocytes with FACS Lysing Solution, cells were fixed and permeabilized with Cytofix/Cytoperm in accordance with the instructions of the manufacturer (BD Pharmingen). Staining of intracytoplasmatic cytokines was performed at 4°C for 45 minutes in the dark with previously determined optimal concentrations of fluorochrome-conjugated monoclonal antibodies for cytokines or appropriate negative (isotype) controls. Besides appropriate isotype controls, an unstimulated sample was included for each patient and control as a negative control.

### Flow cytometric analysis

Multicolor flow cytometric analysis was performed on a FACS Canto II cytometer by using FACSDiva software (BD Biosciences). Lymphocytes were gated for analysis on the basis of light scattering properties and of CD3, CD4, and CD8 staining. Positively and negatively stained populations were calculated by quadrant dot plot analysis determined by isotype controls.

### Statistical analysis

Statistics were performed by using Prism 4.0 (GraphPad Software, La Jolla, CA, USA). Comparisons between patients and control subjects were done by employing the non-parametric Mann-Whitney *U *test. *P *values equal to or less than 0.05 were considered to be statistically significant.

## Results

### Increased frequency of CCR4- and CCR6-expressing CD4^+ ^T cells in granulomatosis with polyangiitis

**Figure 1:**
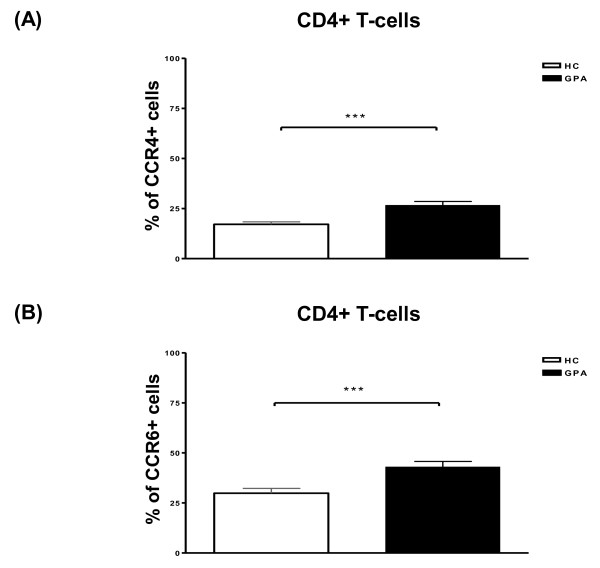
**Increased frequencies of CD4^+^CCR4^+ ^and CD4^+^CCR6^+ ^T cells in patients with granulomatosis with polyangiitis (GPA)**. Percentages of CCR4^+ ^**(a) **and CCR6^+ ^**(b) **cells within the total CD4^+ ^T cell population in patients with GPA and healthy controls (HCs). Percentages of positive cells were assessed by flow cytometry. Values are presented as mean ± standard error of the mean. ***P < 0.001, Mann-Whitney *U *test. CCR, CC chemokine receptor.

To assess CCRs relevant for migration to peripheral tissues, we determined the expression of the CCR4 and CCR6 on peripheral blood T cells in patients with GPA and healthy controls. We found a significant increase in the percentages of CCR4- and CCR6-expressing cells within the total CD4^+ ^T cell population in patients with GPA compared with healthy individuals (Figure 1a, b). Apart from the CCR4^+^CCR6^- ^and CCR4^-^CCR6^+ ^'single positive' subsets, a smaller fraction of CCR4^+^CCR6^+ ^'double positive' cells was detected within the CD4^+ ^T cell population in patients with GPA and healthy controls (17.5% ± 4.8% versus 10.3% ± 0.6%, mean ± standard error of the mean, no significant difference, Mann-Whitney *U *test). Conversely, the remainder of cells within the CD4^+ ^T-cell population were CCR4^-^CCR6^- ^'double negative' cells. In contrast, there was no difference in the percentages of CD8^+^CCR4^+ ^and CD8^+^CCR6^+ ^T cells between patients with GPA and healthy controls (data not shown).

### CCR4 and CCR6 are expressed on distinct memory cell populations, including CCR7^+^CD45RA^med ^very early memory cells

**Figure 2:**
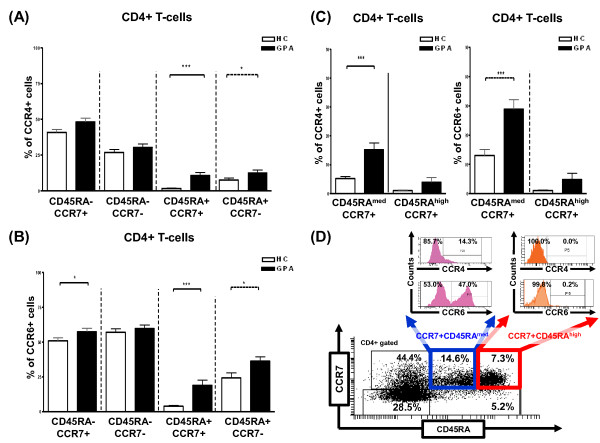
**Increased percentages of CCR4^+ ^and CCR6^+ ^memory T cell subsets in patients with granulomatosis with polyangiitis (GPA)**. Percentages of CD4^+^CCR4^+ ^**(a) **and CD4^+^CCR6^+ ^**(b) **T cells in the CCR7^+^CD45RA^- ^central memory (T_CM_), CCR7^-^CD45RA^- ^effector memory (T_EM_), CCR7^+^CD45RA^+ ^'naïve by definition' (T_N_), and CCR7^-^CD45RA^+ ^'revertant' effector memory (T_EMRA_) populations. **(c) **Dissection of the CD4^+^CCR7^+^CD45RA^+ ^population with regard to CD45RA fluorescence intensity into CCR7^+^CD45RA^high ^cells representing genuine T_N _and CCR7^+^CD45RA^med ^cells reminiscent of very early memory T cells (T_VEM_). Percentages of CCR4^+ ^and CCR6^+ ^cells in the CCR7^+^CD45RA^med ^and CCR7^+^CD45RA^high ^subsets are shown. **(d) **Representative quadrant dot-plot analysis showing segregation of the gated CD4^+^CCR7^+^CD45RA^+ ^T cell population into two subsets. CCR7^+^CD45RA^med ^T cells displayed CCR4 and CCR6 expression (T_VEM_). CCR7^+^CD45RA^high ^T cells lacked CCR4 and CCR6 expression (genuine T_N_). Numbers in quadrants and histograms represent percentages of cells. Percentages of positive cells were assessed by flow cytometry. Values are presented as mean ± standard error of the mean. *P < 0.05, ***P < 0.001, Mann-Whitney *U *test. CCR, CC chemokine receptor; HC, healthy control.

Having shown a significant increase in the frequencies of CCR4- and CCR6-expressing CD4^+ ^T cells in patients with GPA, we were interested in phenotypic features of CCR4- and CCR6-expressing CD4^+ ^T cells next. To assign CCR4^+ ^and CCR6^+ ^cells to the respective naïve and memory cell subsets, cells were additionally stained with fluorescent-conjugated antibodies for CD45RA and CCR7 to allow discrimination into diverse T cell subsets [2,16-18]. By the use of these markers, we found that CCR4 and CCR6 expression was confined largely to the circulating CCR7^+^CD45RA^- ^central memory (T_CM_) and CCR7^-^CD45RA^- ^(T_EM_) and CCR7^-^CD45RA^+ ^(T_EMRA_) effector memory cell subsets within the total CD4^+ ^T cell population. A significant increase in the frequency of CCR4^+ ^and CCR6^+ ^cells was remarkable in the CCR7^-^CD45RA^+ ^effector memory (T_EMRA_) subset in patients with GPA. A significant increase in CCR6^+ ^cells was also found in the CCR7^+^CD45RA^- ^central memory T-cell subset (T_CM_). However, significantly increased percentages of CCR4^+ ^and CCR6^+ ^cells were also detected in the CCR7^+^CD45RA^+ ^population, which contains naïve T cells (T_N_) by definition (Figure 2a, b). Dissecting the CCR7^+^CD45RA^+ ^population with respect to CD45RA fluorescence intensity, we detected two subsets in the CCR7^+^CD45RA^+ ^population. CCR7^+^CD45RA^high ^T cells generally lacked CCR4 and CCR6 expression with the exception of three patients with GPA. In contrast, CCR7^+^CD45RA^med ^T cells displayed CCR4 and CCR6 expression. We found higher frequencies of CCR4^+ ^and CCR6^+ ^cells within the CCR7^+^CD45RA^med ^T cell subset in patients with GPA compared with healthy controls (Figure 2c, d). Thus, the CCR7^+^CD45RA^+ ^population contained genuine CCR7^+^CD45RA^high ^T_N _lacking CCR4 and CCR6 expression and another CCR7^+^CD45RA^med ^T cell subset comprising CCR4^+ ^and CCR6^+ ^cells. The latter was reminiscent of so-called very early memory T cells (T_VEM_) [19].

### Decreased frequency of CCR7^+^CD45RA^high ^naïve T cells and unreduced frequency of CCR7^+^CD45RA^med ^very early memory T cells in granulomatosis with polyangiitis

**Figure 3:**
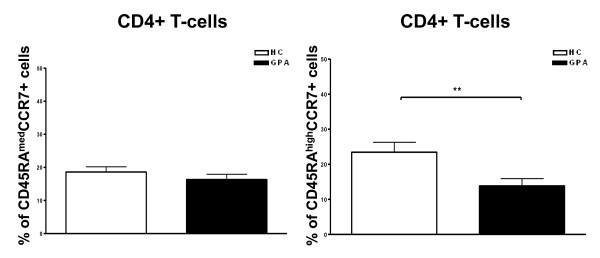
**Decreased frequency of naïve T cells (T_N_) and unreduced frequency of very early memory T cells (T_VEM_) in patients with granulomatosis with polyangiitis (GPA)**. Percentages of CCR7^+^CD45RA^med ^T_VEM _and CCR7^+^CD45RA^high ^T_N _in the CD4^+ ^T cell population are shown. Percentages of positive cells were assessed by flow cytometry. Values are presented as mean ± standard error of the mean. **P < 0.01, Mann-Whitney *U *test. CCR, CC chemokine receptor; HC, healthy control.

Earlier studies have reported significantly lower percentages of peripheral blood T_N _by using CCR7 and CD45RO or CD45RB expression for the phenotypic characterization of T cells in patients with GPA [20,21]. In this study, we showed a segregation of the CCR7^+^CD45RA^+ ^T cell compartment into different two subsets based on CCR4 and CCR6 expression and CD45RA fluorescence intensity. This prompted us to investigate whether T_N _and T_VEM _frequencies were likewise decreased within the total CD4^+ ^T-cell population. In line with the aforementioned earlier studies, we found a significantly lower percentage of CCR7^+^CD45RA^high ^T_N _in patients with GPA compared with healthy controls [20,21]. In contrast, CCR7^+^CD45RA^med ^T_VEM _frequencies were similar in patients with GPA and healthy individuals (Figure 3).

### Distinct cytokine-producing subsets within CCR4^+ ^and CCR6^+ ^T-cell populations

We showed increased frequencies of circulating CCR4- or CCR6-expressing (or both) CD4^+ ^T_CM_, T_EMRA_, and T_VEM _in patients with GPA. To investigate functional features of CCR4^+ ^and CCR6^+ ^cells within the total CD4^+ ^T cell population, peripheral blood cells were stained for intracellular cytokines. An IFNγ^+ ^cell fraction was found in all CCR4^+ ^or CCR6^+ ^subsets or both. An increased percentage of IL-17^+ ^and IL-22^+ ^cells was detected in the CCR4^-^CCR6^+ ^'single positive' and CCR4^+^CCR6^+ ^'double positive' cell fractions when compared with the CCR4^-^CCR6^- ^'double negative' cell subset. Furthermore, an increased frequency of IL-4^+ ^cells was shown in the CCR4^+^CCR6^- ^'single positive' cell fraction compared with the CCR4^-^CCR6^- ^'double negative' cell subset in both patients with GPA and healthy controls. CCR4^-^CCR6^+ ^'single positive' and CCR4^+^CCR6^+ ^'double positive' cells differed from each other with respect to the percentage of IFNγ-producing cells, which was significantly higher in the CCR4-CCR6^+ ^'single positive' fraction (*P *< 0.01, Mann-Whitney *U *test). The CCR4^-^CCR6^- ^'double negative' cell subset differed from the other subsets in that only an IFNγ^+ ^cell fraction was remarkable in this subset (Table 2). Less than 1% of cells displayed intracellular co-expression of IL-17 and IL-22 in CCR6^+ ^cells within the total CD4^+ ^T-cell population (data not shown). Thus, circulating CD4^+^CCR4^+ ^and CD4^+^CCR6^+ ^T cell populations comprised distinct subsets of cytokine-producing cells.

**Table 2:** Percentages of cytokine-producing CCR4^+ ^and/or CCR6^+ ^cell subsets within the total CD4^+ ^T cell population compared with the CCR4^-^CCR6^- ^cell subset

Granulomatosis with polyangiitis				
	CCR4^-^CCR6^- ^cells	CCR4^+^CCR6^- ^cells	CCR4^-^CCR6^+ ^cells	CCR4^+^CCR6^+ ^cells
Percentage of IFNγ^+ ^cells	11.3 ± 5.1	5.5 ± 2.0	28.2 ± 4.3	4.0 ± 0.5
Percentage of IL-4^+ ^cells	1.0 ± 0.5	5.5 ± 1.5^a^	0.6 ± 0.3	1.0 ± 0.4
Percentage of IL-17^+ ^cells	0.1 ± 0.0	0.1 ± 0.0	7.8 ± 1.8^a^	9.5 ± 0.9^b^
Percentage of IL-22^+ ^cells	0.1 ± 0.0	0.2 ± 0.1	2.4 ± 0.5^a^	3.4 ± 0.6^a^
Healthy controls				
	CCR4^-^CCR6^- ^cells	CCR4^+^CCR6^- ^cells	CCR4^-^CCR6^+ ^cells	CCR4^+^CCR6^+ ^cells
Percentage of IFNγ^+ ^cells	21.7 ± 8.4	13.1 ± 2.0	30.7 ± 1.1	6.0 ± 0.5
Percentage of IL-4^+ ^cells	1.0 ± 0.3	5.4 ± 1.0^a^	1.5 ± 0.4	2.2 ± 0.6
Percentage of IL-17^+ ^cells	0.1 ± 0.0	0.2 ± 0.0	9.1 ± 1.0^a^	9.7 ± 1.8^a^
Percentage of IL-22^+ ^cells	0.1 ± 0.0	0.4 ± 0.2	3.3 ± 0.9^a^	5.0 ± 2.1^a^

Percentages of positive cells were assessed by flow cytometry. Values are expressed as mean ± standard error of the mean. ^a^*P *< 0.5, ^b^*P *< 0.01, Mann-Whitney *U *test. CCR, CC chemokine receptor; IFNγ, interferon-gamma; IL, interleukin.

## Discussion

Chemokine receptors play an important role in mediating T cell recruitment to distinct anatomical sites and tissues [2]. Whereas the CC chemokine receptor CCR7 mediates homing of naïve (T_N_) and central memory (T_CM_) T cells to lymph nodes, other CC and CXC chemokine receptors (CCR/CXCR) trigger intravascular adhesion and direct migration of effector memory T cell subsets (CD45RA^- ^T_EM _and CD45RA^+ ^'reverted' T_EMRA_) into peripheral tissues for patrol and recruitment to inflammatory sites [2,19]. Previously, cloned CCR6^+ ^cells from peripheral blood and inflammatory sites in Crohn's disease have been shown to produce IL-17. In contrast, CCR4^+ ^cells secrete IL-4 [11,12,22]. Recently, Th17-, Th22-, and Th2-type PR3-specific cells have been suggested to be involved in chronic inflammation and autoimmunity in GPA [8-10,23]. Moreover, an increased proportion of circulating CD45RC^low ^Th2-type and Th17 cells has been reported in ANCA-associated vasculitides, including GPA. The increase is independent of disease duration and treatment [24]. Therefore, to investigate the extent to which CCR4 and CCR6 expression could be implicated in T-cell recruitment in GPA, we analyzed the expression of these chemokine receptors on T cells.

In this study, we found increased frequencies of circulating CCR4^+ ^and CCR6^+ ^cells within the total CD4^+ ^T cell population in GPA. In contrast, we found no significant increase in the frequencies of CCR4^+ ^and CCR6^+ ^cells in the total CD8^+ ^T cell population. CCR4 and CCR6 expression suggests T cell activation [11,12]. Persistent T cell activation regardless of clinical disease activity has been reported in GPA [20,21,25]. Recently, stable CCR6 expression was reported to be controlled by epigenetic mechanisms [26]. In line with previous reports, CCR4 and CCR6 expression was confined largely to circulating CCR7^+^CD45RA^- ^central memory (T_CM_), CCR7^-^CD45RA^- ^(T_EM_), and CCR7^-^CD45RA^+ ^(T_EMRA_) effector memory CD4^+ ^T cells [11,12]. We found a significant increase in the frequency of CCR4^+ ^and CCR6^+ ^T_EMRA _and CCR6^+ ^T_CM _in patients with GPA. Surprisingly, CCR4^+ ^and CCR6^+ ^cells were also detected within the CCR7^+^CD45RA^+ ^population, which contains the naïve T cell subset (T_N_) by definition. T_N _are CD45RA^+ ^and express CCR7 for peripheral lymph node homing but lack receptors such as CCR4 and CCR6 for the migration to peripheral tissues [2,16-18]. Further analysis dissecting the CCR7^+^CD45RA^+ ^population with regard to CD45RA fluorescence intensity disclosed that the CCR7^+^CD45RA^+ ^T-cell compartment contained two subsets. One subset of CCR7^+^CD45RA^high ^T cells generally lacked CCR4 and CCR6 expression with the exception of three patients with GPA. Thus, CCR7^+^CD45RA^high ^T cells represented genuine T_N_. CCR4 and CCR6 expression on CCR7^+^CD45RA^high ^T_N _in individual patients with GPA could represent T_N _activation, which has been reported before by demonstrating an increased frequency of CD4^+^CD45RO^-^FoxP3^- ^T_N _expressing the activation marker CD25 [20,25]. In line with earlier studies, we showed that the percentage of CCR7^+^CD45RA^high ^T_N _within the total CD4^+ ^T cell population was significantly lower in patients with GPA [20,21]. In contrast, the percentage of CCR7^+^CD45RA^med ^T cells was not decreased in patients with GPA. CCR7^+^CD45RA^med ^T cells displayed CCR4 and CCR6 expression reminiscent of so-called very early memory T cells (T_VEM_). Higher frequencies of CCR4^+ ^and CCR6^+ ^cells within the CCR7^+^CD45RA^med ^T_VEM _subset were found in patients with GPA compared with healthy controls. T_VEM _have been described earlier as 'apparently T_N_' oddly displaying chemokine receptors for both lymph node homing (CCR7) and peripheral tissue migration (CCR4 and CXCR3) in healthy individuals by Song and colleagues [19]. Analysis of the proliferation history, T-cell receptor repertoire, and cytokine response of CCR4- and CXCR3-expressing CCR7^+^CD45RO^- ^T cells suggests that these cells represent T_VEM_, which have proceeded only a short way along the differentiation pathway from T_N _to T_CM _or T_EM_. T_VEM _are still multifunctional but finally differentiate into either T_CM _or T_EM _[19].

Earlier studies showed that chemokine receptor expression for lymph node homing (CCR7) and peripheral tissue migration (for example, CCR4) is not mutually exclusive on T cell subsets [27]. The migratory behavior of T_EM _displaying dual-chemokine receptor expression is determined by chemotactic gradients and cytokine- and T-cell receptor (TCR)-mediated signals [28]. CCR4-expressing CCR7^+ ^T_EM _have been reported in inflamed peripheral tissues (for example, in psoriasis and juvenile idiopathic arthritis) [29,30]. Whereas CCR7^- ^T_EM _remain in the peripheral tissue, CCR7^+ ^T_EM _migrate to peripheral tissues and subsequently exit the tissue to enter draining lymph nodes in different animal models [31,32]. Although CCR7^+ ^T_EM _retain a capability to enter lymph nodes, inflammatory cytokines can subvert migration of CCR7^+ ^T_EM_, resulting in the retention of CCR7^+ ^T_EM _in the inflamed synovial tissue [33]. Cytokines also drive the differentiation of CCR4-expressing CCR7^+ ^T_CM _to CCR7^- ^T_EM _[22]. Of note, CCR7^+ ^T_EM _accumulate in areas of ectopic lymphoid tissue in the inflamed synovial tissue [30]. In contrast, CCR4-expressing CCR7^+ ^T_VEM _reside or recirculate in secondary lymphoid tissues, where they continue to differentiate and acquire further chemokine receptors for peripheral tissue migration [19].

Having shown increased frequencies of circulating CCR4- and CCR6-expressing CD4^+ ^memory T cell subsets, including T_VEM _in patients with GPA, we analyzed the cytokine production of CCR4^+ ^and CCR6^+ ^T cells. Previously, cloned and, as such, preselected CCR6^+ ^cells were reported to secrete IL-17, whereas CCR4^+ ^T cells produce IL-4 [11,12]. In our study, we found an increased percentage of IL-17- and IL-22-producing cells in the CCR4^-^CCR6^+ ^'single positive' and CCR4^+^CCR6^+ ^'double positive' cell subsets and an increased frequency of IL-4^+ ^cells in the CRR4^+^CCR6^- ^'single positive' cell subset compared with the CCR4^-^CCR6^- ^'double negative' cell subset within the total circulating CD4^+ ^T-cell population. Thus, in line with earlier studies, we found Th17 cells within circulating CCR6^+ ^cells and Th2-type cells among CCR4^+ ^cells [11,12]. Moreover, CCR4^-^CCR6^+ ^'single positive' and CCR4^+^CCR6^+ ^'double positive' cells differed from each other with respect to the percentage of IFNγ-producing cells, which was higher in the former cell population.

## Conclusions

We found increased frequencies of circulating CCR4^+ ^and CCR6^+ ^T cells in patients with GPA. CCR4 and CCR6 expression was confined largely to central memory (T_CM_) and effector memory (T_EM _and T_EMRA_) subsets but could also be detected on very early memory T cells (T_VEM_) displaying chemokine receptors for both lymph node homing (CCR7) and peripheral tissue migration (CCR4 and CCR6). CD4^+^CCR4^+ ^and CD4^+^CCR6^+ ^T-cell populations contained distinct cytokine-producing subsets. Our data suggest that CCR4 and CCR6 could be involved in the recruitment of different T cell subsets, including cytokine-producing cells, to inflamed sites in patients with GPA. Further studies are needed to assess CCR4^+ ^and CCR6^+ ^T cell reactivity to the respective chemokine gradients and the expression of CCR4, CCR6, and their chemokine ligands in inflammatory lesions in patients with GPA in order to define new targets for therapeutic intervention.

## Abbreviations

ANCA: anti-neutrophil cytoplasmic autoantibodies; APC: allophycocyanine; APC-Cy7: allophycocyanine-cyanine dye 7; CCR: CC chemokine receptor; CXCR: CXC chemokine receptor; Cy7: cyanine dye 7; FACS: fluorescence-activated cell sorting; GPA: granulomatosis with polyangiitis (Wegener's); IFNγ: interferon-gamma; IL: interleukin; PB: Pacific blue; PE: phycoerythrin; PR3: proteinase 3; T_CM_: central memory T cells; T_EM_: CD45RA^- ^effector memory T cells; T_EMRA_: CD45RA^+ ^effector memory T cells; Th: T-helper cell; T_N_: naïve T cells; T_VEM_: very early memory T cells.

## Competing interests

The authors declare that they have no competing interests.

## Authors' contributions

UF participated in the design of the study, acquisition of data, interpretation of the results, and drafting of the manuscript. SP participated in the acquisition of data, interpretation of results, and drafting of the manuscript. WLG participated in the coordination of the study and assisted in the interpretation of the results. PL conceived the study, participated in its design and coordination and the interpretation of the results, and drafted of the manuscript. All authors read and approved the final manuscript.

## Authors' information

UF, Ph.D., is a biologist. SP is a medical technician. WLG, M.D., is the director of the Department of Rheumatology and spokesman of the Vasculitis Center UKSH and Clinical Research Unit 170. PL, M.D., is the coordinator of Clinical Research Unit 170. All authors are at the Department of Rheumatology, Vasculitis Center UKSH and Clinical Center Bad Bramstedt, University of Lübeck (Lübeck, Germany).
